# Association between daidzein intake and metabolic associated fatty liver disease: A cross-sectional study from NHANES 2017–2018

**DOI:** 10.3389/fnut.2023.1113789

**Published:** 2023-02-13

**Authors:** Zheng Yang, Daoqing Gong, Xinxiang He, Fei Huang, Yi Sun, Qinming Hu

**Affiliations:** ^1^Department of Infectious Disease, Jingzhou Hospital Affiliated to Yangtze University, Jingzhou, China; ^2^Teaching Office, Jingzhou Hospital Affiliated to Yangtze University, Jingzhou, China; ^3^Department of Dermatology, Jingzhou Hospital Affiliated to Yangtze University, Jingzhou, China

**Keywords:** daidzein, MAFLD, NHANES, hepatic steatosis, hepatic fibrosis

## Abstract

**Background:**

Metabolic associated fatty liver disease (MAFLD) has become the most common liver disease globally, yet no new drugs have been approved for clinical treatment. Therefore, we investigated the relationship between dietary intake of soy-derived daidzein and MAFLD, to find potentially effective treatments.

**Methods:**

We conducted a cross-sectional study using data from 1,476 participants in National Health and Nutrition Examination Survey (NHANES) from 2017 to 2018 and their associated daidzein intake from the flavonoid database in the USDA Food and Nutrient Database for Dietary Studies (FNDDS). We investigated the relationship between MAFLD status, controlled attenuation parameter (CAP), AST/Platelet Ratio Index (APRI), Fibrosis-4 Index (FIB-4), liver stiffness measurement (LSM), nonalcoholic fatty liver disease (NAFLD) fibrosis score (NFS), hepatic steatosis index (HSI), fatty liver index (FLI), and daidzein intake by adjusting for confounding variables using binary logistic regression models and linear regression models.

**Results:**

In the multivariable-adjusted model II, there was a negative association between daidzein intake and the incidence of MAFLD (OR for Q4 versus Q1 was 0.65, 95% confidence interval [CI] = 0.46–0.91, *p* = 0.0114, *p* for trend was 0.0190). CAP was also negatively associated with daidzein intake, *β* = −0.37, 95% CI: −0.63 to −0.12, *p* = 0.0046 in model II after adjusting for age, sex, race, marital status, education level, family income-to-poverty ratio (PIR), smoking, and alcohol consumption. Stratified by quartiles of daidzein intake, trend analysis of the relationship between daidzein intake and CAP remained significant (*p* for trend = 0.0054). In addition, we also found that HSI, FLI, and NFS were negatively correlated with daidzein intake. LSM was negatively related to daidzein intake but had no statistical significance. The correlation between APRI, FIB-4, and daidzein intake was not strong (although *p* < 0.05, β values were all 0).

**Conclusion:**

We found that MAFLD prevalence, CAP, HSI, and FLI, all decreased with increased daidzein intake, suggesting that daidzein intake may improve hepatic steatosis. Therefore, dietary patterns of soy food or supplement consumption may be a valuable strategy to reduce the disease burden and the prevalence of MAFLD.

## Introduction

Chronic liver disease is the leading cause of both morbidity and mortality worldwide, with 2 million deaths from the liver disease each year (1, 2). With the improvement of people’s living conditions, dietary patterns, and lifestyle changes, the incidence of non-alcoholic fatty liver disease (NAFLD) has been on the rise year by year and is now the fastest-growing cause of cirrhosis and liver cancer incidence and its associated mortality, resulting in a serious clinical and socio-economic burden (3–5). NAFLD has become the most prevalent chronic liver disease worldwide in the last two decades, and affects about a quarter of the world’s population (6). In addition to liver involvement, NAFLD is associated with an increased risk of diabetes, cardiovascular disease, cerebrovascular disease, chronic kidney disease, and extrahepatic tumors (7). In 2020, the international consensus proposed a new concept: metabolic associated fatty liver disease (MAFLD), which emphasized the role of metabolic disorders without excluding patients with an intake of alcohol or other chronic liver diseases and differed significantly from the diagnostic criteria for NAFLD (8, 9). In the real world, MAFLD diagnostic criteria are more practical than NAFLD diagnostic criteria for recognizing patients with a fatty liver at a high risk of progressive disease (10). The use of MAFLD criteria is more helpful in identifying and treating fatty liver patients at risk of hepatic fibrosis through non-invasive tests (11, 12). However, the treatment of NAFLD/MAFLD is limited to lifestyle changes, and there is a lack of clear and effective drugs (13).

Current studies have confirmed that the macronutrient and micronutrient composition of food can promote or prevent MAFLD (14–16). MAFLD is considered to be the liver manifestation of metabolic syndrome, and its occurrence is usually associated with chronic exposure to a nutrient-deficient diet (16). Wang et al. (17) showed that flavonoids can block oxidative stress by inhibiting CYP2E1 activity, thus improving insulin resistance, lipid peroxidation, and endoplasmic reticulum stress to prevent and treat NAFLD. Flavonoids are natural polyphenolic compounds that are widely found in plants and can be classified into several subtypes based on the degree of oxidation, mainly including isoflavones, flavonoids, flavanones, flavanols, flavonols, and flavan-3-ols (18). Among them, isoflavone compounds belong to phytoestrogens, mainly including daidzein, glycitein, genistein, biochanin A and formononetin (19). Equol is not a phytoestrogen, but as a metabolite of daidzein, it is sometimes included in the isoflavone class (20). The main dietary sources of human isoflavones are soybeans and soy products, containing daidzein and genistein (21). The chemical structure of daidzein is similar to that of mammalian estrogens and can act by replacing or blocking this hormone and its corresponding receptors, thus making daidzein a drug candidate with dual use (22). This makes daidzein a potential therapeutic option in estrogen-dependent diseases like prostate cancer, breast cancer, diabetes, and cardiovascular disease (23–25). Yamagata et al. (26) showed that daidzein had the potential to prevent metabolic syndrome by affecting hypertension, hyperglycemia, dyslipidemia, and atherosclerosis in patients. Another research surveyed the relationship between dietary isoflavone intake and risk of metabolic disorders in 6786 Chinese adults, which showed that total isoflavones, genistein, and daidzein intake were all negatively associated with NAFLD, hyperlipidemia, and hypertension (27). However, it is necessary for us to study the role of daidzein in MAFLD of different ethnicities. In this study, we prospectively surveyed the relationship between dietary daidzein intake and the incidence of MAFLD, hepatic steatosis, and hepatic fibrosis in participants in the National Health and Nutrition Examination Survey (NHANES) to find a potentially effective treatment for MAFLD.

## Materials and methods

### Study population

The National Health and Nutrition Examination Survey (NHANES) is a continuous cross-sectional sample survey conducted since 1999 by the National Center for Health Statistics of the U.S. Centers for Disease Control and Prevention, every 2 years representing a survey cycle. The project is a complex, nationally representative, stratified, multi-stage probabilistic health survey of the non-institutionalized civilian population of the United States. The specific data collection procedures and detailed methodology for NHANES have been described by the National Center for Health Statistics (28). NHANES data are obtained from interviews, laboratory tests, and physical examinations conducted by trained staff. In our study, we specifically used data from one cycle of NHANES 2017–2018, which evaluated parameters of hepatic steatosis and fibrosis in patients using vibration-controlled transient elastography (VCTE) (29). Flavonoid intake data for patients in this study are derived from the 2017–2018 Food and Beverage Survey flavonoid values in the NHANES-associated USDA Food and Nutrient Database for Dietary Studies (FNDDS). There are more than 7,000 foods/beverages with flavonoid values in FNDDS 2017–2018, which can calculate the estimated value of flavonoid intake representing the United States population of all ages. Linking these estimates to laboratory data, physical examination, and interview data in NHANES can better investigate the relationship between flavonoid intake and human health (30). The data and documents used in this study are unidentified data frames that are publicly available on the National Center for Health Statistics (31) and Agricultural Research Service (30) websites. Ethics approval has been granted by the NCHS Ethics Review Committee, and protocol descriptions are available at.^1^ Written informed consent is required for participants 12 and over, and parental consent is also required for participants under 18 years old.

**Figure 1:**
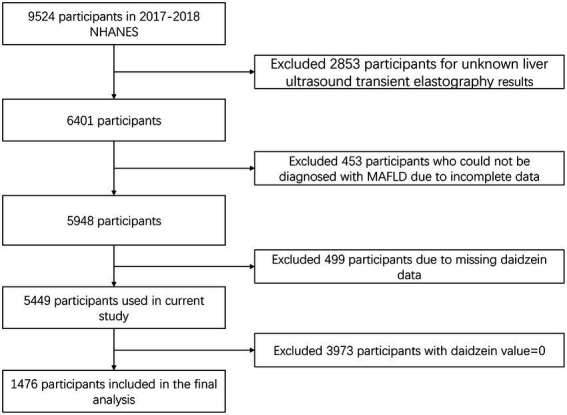
Flowchart of the selection process of the participants in NHANES 2017–2018. NHANES, National Health and Nutrition Examination Survey; MAFLD, metabolic associated fatty liver disease.

A total of 9,524 participants were recruited into the study during the 2017–2018 NHANES cycle. After excluding 2,853 participants who were not assessed for hepatic steatosis and liver fibrosis using VCTE at baseline, 453 participants who could not be diagnosed with MAFLD due to incomplete information, 499 participants with no daidzein value, and 3,973 participants with a daidzein value of 0, a total of 1,476 participants were finally enrolled in the analysis (Figure 1).

### Evaluation of daidzein intake

The flavonoid database in the FNDDS provides the total intake of 29 individual flavonoids, the total of the 6 major flavonoids, and the total intake of all flavonoids consumed by each participant on day 1 and day 2, respectively. Isoflavones are mainly sourced from soy foods/beverages such as soy milk, soy-based proteinaceous powder, and tofu, as well as isoflavone ingredients that are added to foods/beverages as additives to achieve specific functions. The 2017–2018 FNDDS nutrient values were calculated for each food/beverage based on the ingredient data in FoodData Central (32). In this study, we selected the mean of daidzein intake on days 1 and 2 for each participant in the flavonoid database to investigate its association with MAFLD incidence, hepatic steatosis, and hepatic fibrosis.

### Definition of MAFLD

The diagnosis of MAFLD in this study was by the VCTE measurement of hepatic steatosis and the presence of one of the following three conditions, such as overweight/obesity, diabetes mellitus (DM), or evidence of metabolic abnormalities (9). Evidence of metabolic abnormalities was defined as at least 2 of the following metabolic risk abnormalities such as (1) waist circumference ≥102 and 88 cm in men and women, respectively, (2) blood pressure ≥ 130/85 mm Hg or treated with specific medications, (3) plasma triglycerides (TG) ≥1.70 mmol/l or treated with specific medications, (4) high-density lipoprotein cholesterol (HDL-C) <1.0 mmol/l in men and <1.3 mmol/l in women or treated with specific medications, (5) prodromal diabetes (fasting glucose) level 5.6–6.9 mmol/l or hemoglobin A1c 5.7–6.4%, (6) insulin resistance score ≥2.5 as assessed by the homeostatic model, (7) plasma high-sensitivity C-reactive protein level >2 mg/l.

### Non-invasive evaluation of liver disease

During the 2017–2018 NHANES cycle, the technicians performed VCTE (Echosens, Paris, France) tests using the FibroScan 502 V2 Touch model after 2 days of professional training and authorization following the liver ultrasound transient elastography operation manual (33). Technicians obtained at least 10 measurements from participants who fasted for at least 3 h, yielding median controlled attenuation parameter (CAP) and liver stiffness measurement (LSM) values and interquartile spacing for each participant. The higher the CAP value measured, the higher the liver fat content (CAP reference range: 100–400 dB/m); similarly, the higher the LSM value, the more severe the hepatic fibrosis (LSM reference range: 1.5–75 kPa). In this study, we defined CAP ≥248 dB/m as hepatic steatosis according to the published data of a large meta-analysis (34) and the findings of a study of hepatic steatosis in an adolescent population (35). Eddowes et al. (36) showed cutoff values of 8.2 kPa, 9.7 kPa, and 13.6 kPa for F > F2, F > F3, and F=F4, respectively; therefore, we defined progressive hepatic fibrosis as LSM ≥ 9.7 KPa.

In the study, we also used serum-based scores for the assessment of non-invasive hepatic steatosis and liver fibrosis to investigate their relationship with daidzein intake. Liver steatosis score includes Fatty liver index (FLI) (FLI is calculated based on TG, gamma glutamyl transferase (GGT), body mass index (BMI), and waist circumference; FLI ≥ 60 is judged as hepatic steatosis) (37) and Hepatic steatosis index (HSI) (calculated based on alanine aminotransferase (ALT), aspartate aminotransferase (AST), BMI, diabetes mellitus (DM), gender; HSI > 36 is judged as NAFLD) (38). Hepatic fibrosis scores include AST to platelet ratio index (APRI) (results based on AST and platelet (PLT) count, APRI >1.5 indicates significant liver fibrosis) (39), Fibrosis-4 (FIB-4) (results based on ALT, AST, PLT and age, FIB-4 > 2.67 suggests that there are NAFLD patients with grade F3-4 or higher hepatic fibrosis) (40, 41) and NAFLD fibrosis score (NFS) (NFS calculated based on albumin, ALT, AST, PLT count, age, DM or impaired fasting blood glucose, and BMI, with NFS > 0.676 suggesting that NAFLD patients have progressive hepatic fibrosis) (42); the above scoring models of formulae for calculation are shown in Supplementary Table S1.

### Covariates

Demographic, physical examination, and laboratory test information in this study are available from the NHANES database, which mainly include age, sex, race, marital status, education level, economic status, BMI, smoking, drinking, homeostasis model assessment of insulin resistance (HOMA-IR), waist circumference and history of hypertension and diabetes. BMI = weight [kg]/height [m^2^], classified as <25, 25–30, and ≥30 (43); HOMA-IR is fasting insulin (μU/mL) × fasting glucose (mmol/L)/22.5 (44). Hypertension is defined as systolic blood pressure (SBP) ≥ 140 mmHg and diastolic blood pressure (DBP) ≥90 mmHg or the use of antihypertensive drugs. The diagnosis of diabetes mellitus is defined as a self-reported physician diagnosis of diabetes mellitus and/or fasting glucose ≥7.0 mmol/l or glycosylated hemoglobin (HbA1c) ≥6.5% and/or taking diabetes medications (45). We defined current smokers as individuals who have smoked more than 100 cigarettes in their lives and currently smoke on some days or every day; never smokers as people who have smoked less than 100 cigarettes in their lives; and former smokers as people who have smoked more than 100 cigarettes in their lives and now do not smoke at all. We assessed drinking status according to the volume and frequency of alcohol consumption in participants’ self-report questionnaires (46). The races are classified as non-Hispanic white, non-Hispanic black, Mexican-American, and other races. Marital status is divided into married/cohabiting and unmarried. Education level is classified as below high school, high school or the equivalent, and more than high school. Economic status is evaluated by the family income-to-poverty ratio (PIR), which is categorized as <1.0, 1.0–3.0, and >3.0.

Laboratory tests in this study included fasting glucose, fasting insulin, HbA1c, total bilirubin (TBIL), AST, ALT, GGT, alkaline phosphatase (ALP), albumin, creatinine, TG, uric acid, HDL-C, total cholesterol (TC), lactate dehydrogenase (LDH), high sensitivity-C-reactive protein (Hs-CRP), hemoglobin (Hb), white blood cell (WBC) counts, and red blood cell (RBC) count. All routine biochemical tests were performed according to NHANES laboratory/medical technician procedure manual standards (47).

### Statistical analysis

All statistical analyses in this study were performed using R software (version 4.2.0^2^) and Empower Stats software (^3^X&Y Solutions Inc.). The level of significance of the reported statistical results for all analyses was two-tailed, and *p* < 0.05 was considered statistically significant. Missing values in the study were processed by directly deleting the participant data if the exposure factor daidzein intake or the outcome indicator MAFLD was missing. The missing values of other confounders are processed by random forest interpolation using the “missForest” R package. In the analysis of participant baseline characteristics, we divided the exposure factor daidzein intake into quartiles and compared statistical differences between quartile groups. We used weighted linear regression analysis to calculate *p* values for continuous variables, while p values for categorical variables were calculated using a weighted chi-square test. Generalized additive models (GAM) and smoothing curve fitting were used to study whether the exposure variables were nonlinearly related to the outcome variables. The inflection points of the smoothed curves were analyzed by saturation and threshold effects, and the inflection points were calculated by two-stage linear regression analysis. We investigated the relationship between MAFLD status and daidzein intake by multivariate binary logistic regression models, and the relationship between APRI, CAP, LSM, FIB-4, NFS, FLI, HSI, and daidzein intake were assessed, respectively, using multivariate linear regression models. In this study, we constructed three models: crude model: no adjustment for any covariates; model I: adjusted for age, sex, and race; model II: adjusted for covariates of age, sex, race, marital status, education level, PIR, smoking, and alcohol consumption. In addition, we conducted subgroup analyses stratified by variables of interest and used the “forestlater” R package to draw forest plots to show the results of the subgroup analyses.

## Results

A total of 1,476 participants were included in this study, with a weighted mean age of 45.24 ± 18.28 years and a weighted ratio of 50% for both males and females. The median level of daidzein intake in our study was 0.18 mg (interquartile range [IQR], 0.02–1.55 mg). Table 1 described the baseline characteristics of participants by daidzein level quartiles. Laboratory findings of participants by quartiles of daidzein intake are shown in Supplementary Table S2. The findings suggest that participants in the third quartile may be younger compared with those in the first quartile of daidzein intake levels (49.00 ± 17.79 years vs. 41.49 ± 19.00 years) and that the daidzein diet intake population concentrated between the ages of 20–65 years. The proportion of daidzein intake was significantly higher among non-Hispanic whites, those with a high school degree or more, those married/cohabiting, and those with higher household income (PIR > 3, i.e., household income above three times the poverty line) than among other populations [e.g., other Hispanics, those below high school level, those unmarried, and those with lower household income (PIR < 1)]. In addition, the proportion of daidzein intake was significantly higher in participants without pre-diabetes, diabetes, and hypertension compared to those with underlying conditions such as these conditions.

**Table 1:** Baseline characteristics of participants by quartiles of daidzein intake (*N* = 1,476).

Characteristic	Quartile 1	Quartile 2	Quartile 3	Quartile 4	value of *p*
	<0.02	≥0.02 to <0.18	≥0.18 to <1.55	≥1.55	
No. of participants	290	425	377	384	
Age (years)	49.00 ± 17.79	44.42 ± 17.60	41.49 ± 19.00	46.43 ± 17.97	<0.0001
**Age group (%)**					<0.0001
> = 12, <20	4.49	8.85	15.66	5.46	
> = 20, <65	72.25	78.78	71.28	75.59	
> = 65	23.26	12.36	13.06	18.95	
**Gender (%)**					0.0056
Female	50.34	48.77	43.50	56.00	
Male	49.66	51.23	56.50	44.00	
**Race/ethnicity (%)**					0.0001
Non-Hispanic white	66.70	58.50	60.48	65.23	
Non-Hispanic black	11.79	7.44	11.18	4.88	
Other race - including multi-racial	8.42	19.99	11.86	15.86	
Mexican American	7.63	7.99	9.71	6.43	
Other Hispanic	5.46	6.08	6.76	7.60	
**Education level (%)**					0.0005
Less than high school	11.61	13.50	19.22	9.51	
High school or equivalent	22.00	19.67	25.83	22.42	
Above high school	66.39	66.83	54.94	68.07	
**Marital status (%)**					0.0111
Married/cohabiting	63.98	64.62	55.65	66.58	
Unmarried	36.02	35.38	44.35	33.42	
**PIR (%)**					<0.0001
PIR < 1	9.58	7.93	17.74	6.75	
1 < =PIR < =3	33.85	31.62	33.73	30.95	
PIR > 3	56.57	60.44	48.54	62.30	
**Smoking status (%)**					0.0006
Never	52.81	66.17	66.93	64.19	
Past/current	47.19	33.83	33.07	35.81	
**Alcohol consumption (%)**					<0.0001
Never	7.10	11.88	14.99	10.25	
Mild	63.41	49.50	43.95	51.52	
Moderate	13.28	16.14	18.22	21.71	
Heavy	16.21	22.48	22.84	16.52	
Waist circumference (cm)	97.03 ± 16.67	96.28 ± 17.31	98.78 ± 19.35	96.85 ± 18.50	0.2760
**BMI (kg/m** ^**2**^ **)**	28.01 ± 6.68	28.37 ± 7.20	29.23 ± 7.49	28.28 ± 7.29	0.1372
BMI categories (%)					0.0131
BMI < 25	32.46	34.93	33.38	36.74	
25 < =BMI < 30	36.18	33.18	24.83	28.93	
BMI > =30	31.36	31.89	41.79	34.33	
**HOMA_IR**	4.10 ± 5.95	3.29 ± 3.24	3.91 ± 3.48	3.85 ± 8.97	0.3145
preDM (%)					0.0210
No	47.51	55.22	54.31	56.48	
preDM	33.60	32.90	32.46	33.76	
DM	18.89	11.88	13.23	9.76	
**DM (%)**					<0.0001
No	77.63	77.58	78.69	85.31	
IFG	3.48	10.54	8.09	4.93	
DM	18.89	11.88	13.23	9.76	
**Hypertension (%)**					0.1359
No	52.28	61.14	56.89	55.99	
Yes	47.72	38.86	43.11	44.01	
**NFS**	−1.75 ± 1.53	−1.84 ± 1.43	−1.97 ± 1.66	−1.85 ± 1.35	0.3104
NFS categories (%)					0.0446
NFS < −1.455	59.06	64.36	64.14	63.25	
−1.455 = <NFS < 0.676	35.88	30.63	27.37	32.83	
NFS > =0.676	5.06	5.01	8.49	3.92	
**FIB-4**	1.04 ± 0.66	0.93 ± 0.51	0.86 ± 0.58	1.02 ± 0.62	0.0001
FIB-4 categories (%)					0.0002
FIB-4 < 1.3	72.90	82.09	84.10	73.08	
1.3 < =FIB-4 < 2.67	24.10	17.08	14.30	25.63	
FIB-4 > =2.67	3.00	0.83	1.60	1.29	
**FLI**	47.58 ± 32.53	45.39 ± 32.54	47.83 ± 34.81	44.62 ± 30.87	0.4451
FLI categories (%)					0.1675
FLI < 60	60.27	62.17	57.81	65.36	
FLI > =60	39.73	37.83	42.19	34.64	
**HSI**	36.97 ± 8.42	36.60 ± 9.48	38.03 ± 9.52	36.70 ± 9.22	0.1344
HSI categories (%)					0.7269
HSI < 36	53.27	52.88	49.33	51.40	
HSI > =36	46.73	47.12	50.67	48.60	
**LSM (kPa)**	5.47 ± 3.90	5.39 ± 4.29	5.65 ± 4.39	5.39 ± 3.67	0.7917
LSM categories (%)					0.5451
LSM < 9.7	95.95	95.91	93.97	95.65	
LSM > =9.7	4.05	4.09	6.03	4.35	
**CAP (dB/m)**	259.86 ± 58.60	255.28 ± 65.48	258.84 ± 64.57	246.30 ± 59.99	0.0091
CAP categories (%)					0.2531
CAP<248	46.00	52.08	50.23	53.25	
CAP> = 248	54.00	47.92	49.77	46.75	
**APRI**	0.32 ± 0.22	0.33 ± 0.18	0.31 ± 0.17	0.35 ± 0.26	0.1212
APRI categories (%)					0.2619
< = 0.5	93.62	92.18	91.34	89.90	
>0.5, < = 1.5	5.95	7.47	8.62	8.95	
>1.5	0.42	0.35	0.05	1.15	

Means of continuous variables (±SD): value of *p* calculated by weighted linear regression. Percentages of categorical variables: value of *p* calculated by the weighted Chi-square test. Statistical significance was set for a value of *p* < 0.05. PIR, family income-to-poverty ratio; BMI, body mass index; HOMA-IR, homeostasis model assessment of insulin resistance; preDM, prediabetes; DM, diabetes mellitus; IFG, impaired fasting glucose; NFS, NAFLD fibrosis score; FIB-4, fibrosis-4; FLI, fatty liver index; HSI, hepatic steatosis index; LSM, liver stiffness measurement; CAP, controlled attenuation parameter; APRI, AST to platelet ratio index.

**Figure 2:**
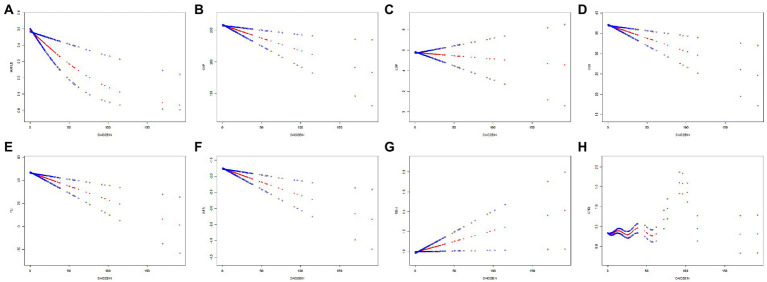
The relationship between daidzein intake and MAFLD prevalence, CAP, LSM, HSI, FLI, NFS, FIB-4, and APRI, respectively. The generalized additive model (GAM) was used to assess the relationship between daidzein intake and MAFLD prevalence **(A)**, CAP **(B)**, LSM **(C)**, HSI **(D)**, FLI **(E)**, NFS **(F)**, FIB-4 **(G)**, and APRI **(H)**, respectively. The red dashed line represents the smoothed curve fit between the variables. The blue dashed line represents the 95% confidence interval of the fit. Age, sex, and race/ethnicity were adjusted in the model. MAFLD, metabolic associated fatty liver disease; CAP, controlled attenuation parameter; LSM, liver stiffness measurement; HSI, hepatic steatosis index; FLI, fatty liver index; FIB-4, fibrosis-4; NFS, NAFLD fibrosis score; APRI, AST to platelet ratio index.

**Figure 3:**
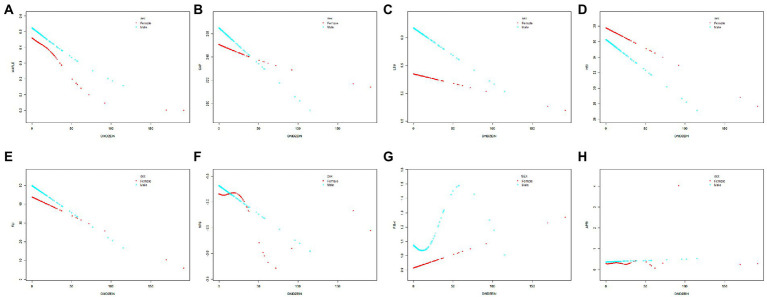
The relationship between daidzein intake and MAFLD prevalence, CAP, LSM, HSI, FLI, NFS, FIB-4, and APRI stratified by gender, respectively. The relationship between daidzein intake and MAFLD prevalence **(A)**, CAP **(B)**, LSM **(C)**, HSI **(D)**, FLI **(E)**, NFS **(F)**, FIB-4 **(G)**, and APRI **(H)** stratified by gender, respectively. Age and race/ethnicity were adjusted in the model. MAFLD, metabolic associated fatty liver disease; CAP, controlled attenuation parameter; LSM, liver stiffness measurement; HSI, hepatic steatosis index; FLI, fatty liver index; FIB-4, fibrosis-4; NFS, NAFLD fibrosis score; APRI, AST to platelet ratio index.

Table 2 showed the relationship between MAFLD incidence and daidzein intake evaluated by three univariate and multivariate binary logistic regression models. In the crude model, there was a negative linear correlation between daidzein intake and MAFLD incidence (OR for Q4 versus Q1 was 0.63, 95% CI: 0.46–0.86, *p* for trend was 0.0024). Multivariate adjusted model II also revealed a significant negative association between daidzein intake and the incidence of MAFLD with an OR of 0.98 (0.97, 0.99) and a value of *p* of 0.0039. Similarly, participants in the highest quartile had an OR of 0.65, 95% CI: 0.46–0.86, compared to those in the lowest quartile of daidzein intake, and statistical significance remained. In addition, we observed a dose–response relationship between daidzein intake and MAFLD incidence, and MAFLD incidence decreased with increasing daidzein intake independent of gender (Figures 2A, 3A; Table 3).

**Table 2:** Relationship between MAFLD prevalence, APRI, CAP, LSM, FIB-4, NFS, FLI, HSI and daidzein intake in a multiple regression model.

Outcome	Crude model	Model I	Model II
**Daidzein (MAFLD)**	0.99 (0.98, 1.00) 0.0227	0.98 (0.97, 0.99) 0.0026	0.98 (0.97, 0.99) 0.0039
Q1	Ref	Ref	Ref
Q2	0.82 (0.61, 1.11) 0.1927	0.92 (0.67, 1.27) 0.6132	0.88 (0.64, 1.23) 0.4576
Q3	0.73 (0.53, 0.99) 0.0411	0.96 (0.69, 1.33) 0.7985	0.96 (0.69, 1.35) 0.8194
Q4	0.63 (0.46, 0.86) 0.0033	0.68 (0.49, 0.94) 0.0212	0.65 (0.46, 0.91) 0.0114
*p* for trend	0.86 (0.78, 0.95) 0.0024	0.89 (0.80, 0.99) 0.0262	0.88 (0.80, 0.98) 0.0190
**Daidzein (CAP)**	−0.33 (−0.60, −0.06) 0.0181	−0.40 (−0.66, −0.14) 0.0024	−0.37 (−0.63, −0.12) 0.0046
Q1	Ref	Ref	Ref
Q2	−7.85 (−17.15, 1.45) 0.0983	−5.58 (−14.53, 3.37) 0.2216	−6.40 (−15.35, 2.56) 0.1617
Q3	−10.06 (−19.60, −0.52) 0.0390	−2.27 (−11.41, 6.88) 0.6271	−1.91 (−11.07, 7.26) 0.6836
Q4	−16.51 (−26.01, −7.00) 0.0007	−14.06 (−23.16, −4.96) 0.0025	−14.95 (−24.07, −5.83) 0.0013
*p* for trend	−5.08 (−8.03, −2.12) 0.0008	−3.89 (−6.71, −1.07) 0.0069	−4.02 (−6.85, −1.20) 0.0054
**Daidzein (LSM)**	−0.01 (−0.03, 0.01) 0.5342	−0.01 (−0.03, 0.01) 0.5535	−0.01 (−0.03, 0.01) 0.5171
Q1	Ref	Ref	Ref
Q2	−0.24 (−0.96, 0.47) 0.5081	−0.03 (−0.76, 0.69) 0.9289	−0.07 (−0.80, 0.66) 0.8529
Q3	0.07 (−0.66, 0.81) 0.8516	0.33 (−0.41, 1.07) 0.3811	0.29 (−0.45, 1.04) 0.4375
Q4	−0.62 (−1.35, 0.12) 0.0995	−0.39 (−1.12, 0.35) 0.3026	−0.42 (−1.16, 0.31) 0.2604
*p* for trend	−0.16 (−0.38, 0.07) 0.1818	−0.09 (−0.32, 0.14) 0.4469	−0.10 (−0.33, 0.13) 0.3988
**Daidzein (HSI)**	−0.06 (−0.10, −0.02) 0.0024	−0.06 (−0.10, −0.03) 0.0012	−0.06 (−0.10, −0.02) 0.0027
Q1	Ref	Ref	Ref
Q2	−0.80 (−2.17, 0.57) 0.2527	−0.02 (−1.38, 1.34) 0.9771	−0.19 (−1.53, 1.15) 0.7846
Q3	−0.19 (−1.60, 1.22) 0.7907	0.66 (−0.73, 2.05) 0.3537	0.76 (−0.61, 2.13) 0.2760
Q4	−1.12 (−2.52, 0.29) 0.1188	−0.51 (−1.90, 0.87) 0.4691	−0.72 (−2.09, 0.64) 0.2979
*p* for trend	−0.26 (−0.70, 0.18) 0.2448	−0.10 (−0.53, 0.33) 0.6483	−0.13 (−0.56, 0.29) 0.5356
**Daidzein (FLI)**	−0.20 (−0.34, −0.06) 0.0048	−0.24 (−0.37, −0.11) 0.0003	−0.22 (−0.35, −0.09) 0.0008
Q1	Ref	Ref	Ref
Q2	−3.69 (−8.49, 1.10) 0.1314	0.79 (−3.70, 5.29) 0.7292	0.16 (−4.28, 4.60) 0.9431
Q3	−3.21 (−8.13, 1.70) 0.2004	2.59 (−2.00, 7.18) 0.2686	2.70 (−1.84, 7.24) 0.2444
Q4	−6.28 (−11.18, −1.38) 0.0121	−2.67 (−7.24, 1.90) 0.2526	−3.35 (−7.87, 1.17) 0.1462
*p* for trend	−1.77 (−3.30, −0.25) 0.0227	−0.73 (−2.15, 0.68) 0.3113	−0.85 (−2.25, 0.55) 0.2354
**Daidzein (FIB-4)**	0.00 (0.00, 0.01) 0.0075	0.00 (0.00, 0.00) 0.0367	0.00 (0.00, 0.00) 0.0441
Q1	Ref	Ref	Ref
Q2	−0.15 (−0.25, −0.05) 0.0037	−0.02 (−0.09, 0.05) 0.6435	−0.01 (−0.09, 0.06) 0.7107
Q3	−0.27 (−0.38, −0.17) <0.0001	−0.02 (−0.10, 0.05) 0.5368	−0.03 (−0.10, 0.05) 0.4690
Q4	−0.13 (−0.23, −0.02) 0.0155	−0.01 (−0.08, 0.06) 0.7749	−0.01 (−0.08, 0.07) 0.8690
*p* for trend	−0.04 (−0.08, −0.01) 0.0076	−0.00 (−0.03, 0.02) 0.7913	−0.00 (−0.03, 0.02) 0.8301
**Daidzein (NFS)**	−0.00 (−0.01, 0.00) 0.2792	−0.01 (−0.01, −0.00) 0.0005	−0.01 (−0.01, −0.00) 0.0006
Q1	Ref	Ref	Ref
Q2	−0.25 (−0.49, −0.01) 0.0435	0.13 (−0.03, 0.30) 0.1038	0.13 (−0.04, 0.29) 0.1291
Q3	−0.52 (−0.77, −0.28) <0.0001	0.10 (−0.06, 0.27) 0.2279	0.10 (−0.07, 0.26) 0.2434
Q4	−0.36 (−0.61, −0.11) 0.0048	−0.02 (−0.19, 0.14) 0.7698	−0.03 (−0.20, 0.13) 0.7016
*p* for trend	−0.13 (−0.20, −0.05) 0.0013	−0.02 (−0.07, 0.03) 0.4811	−0.02 (−0.07, 0.03) 0.4454
**Daidzein (APRI)**	0.00 (0.00, 0.00) <0.0001	0.00 (0.00, 0.00) <0.0001	0.00 (0.00, 0.00) <0.0001
Q1	Ref	Ref	Ref
Q2	−0.01 (−0.05, 0.03) 0.5089	−0.01 (−0.04, 0.03) 0.7851	−0.00 (−0.04, 0.04) 0.8813
Q3	−0.03 (−0.07, 0.01) 0.1099	−0.02 (−0.06, 0.02) 0.2664	−0.02 (−0.06, 0.02) 0.2764
Q4	−0.00 (−0.04, 0.04) 0.8794	0.00 (−0.03, 0.04) 0.8462	0.01 (−0.03, 0.05) 0.7468
*p* for trend	−0.00 (−0.01, 0.01) 0.7374	−0.00 (−0.01, 0.01) 0.9951	0.00 (−0.01, 0.01) 0.9395

Crude model: There are no covariates were adjusted. Model I: Age, sex, and race/ethnicity were adjusted. Model II: Age, sex, and race/ethnicity, marital status, education level, PIR, smoking, and alcohol use were adjusted. MAFLD, metabolic associated fatty liver disease; Q, quartile of daidzein; PIR, family income-to-poverty ratio; CAP, controlled attenuation parameter; LSM, liver stiffness measurement; HSI, hepatic steatosis index; FLI, fatty liver index; FIB-4, fibrosis-4; NFS, NAFLD fibrosis score; APRI, AST to platelet ratio index; Ref, reference.

**Table 3:** Threshold effect analysis of daidzein intake using two-segment linear regression models for MAFLD prevalence, APRI, CAP, LSM, FIB-4, NFS, FLI, and HSI, respectively.

Outcome	MAFLD	CAP	LSM	HSI	FLI	FIB-4	NFS	APRI*	APRI**
Model I: Fitting by the standard linear model	1.0 (1.0, 1.0) 0.003	−0.4 (−0.7, −0.1) 0.002	−0.0 (−0.0, 0.0) 0.554	−0.1 (−0.1, −0.0) 0.001	−0.2 (−0.4, −0.1) <0.001	0.0 (0.0, 0.0) 0.037	−0.0 (−0.0, −0.0) <0.001	0.0 (0.0, 0.0) <0.001	0.0 (0.0, 0.0) <0.001
*P* interaction	0.603	0.136	0.760	0.506	0.493	0.710	0.310	0.102	0.102
**Model II: Fitting by the two-piecewise linear model**									
Inflection point (K)	2.2	2.9	2.3	0	0	0.1	0	0.3	0.4
<K-segment effect 1	0.9 (0.8, 1.0) 0.096	−3.3 (−6.2, −0.4) 0.028	−0.2 (−0.5, 0.1) 0.178	NA	NA	−0.4 (−1.0, 0.2) 0.210	NA	−0.1 (−0.2, −0.0) 0.047	0.7 (−0.8, 2.1) 0.391
>K-segment effect 2	1.0 (1.0, 1.0) 0.040	−0.3 (−0.6, 0.0) 0.082	0.0 (−0.0, 0.0) 0.944	−0.1 (−0.1, −0.0) 0.001	−0.2 (−0.4, −0.1) <0.001	0.0 (0.0, 0.0) 0.020	−0.0 (−0.0, −0.0) <0.001	0.0 (0.0, 0.0) <0.001	0.0 (0.0, 0.0) <0.001
Effect difference between 2 and 1	1.1 (1.0, 1.3) 0.156	3.0 (−0.0, 6.1) 0.053	0.2 (−0.1, 0.5) 0.191	NA	NA	0.4 (−0.2, 1.0) 0.207	NA	0.1 (0.0, 0.2) 0.043	−0.6 (−2.1, 0.8) 0.393
Predicted value of the equation at the folding point	−0.3 (−0.5, −0.1)	248.9 (241.6, 256.2)	5.3 (4.8, 5.9)	37.0 (36.5, 37.5)	46.7 (45.0, 48.4)	0.9 (0.9, 0.9)	−1.8 (−1.9, −1.7)	0.3 (0.3, 0.3)	0.3 (0.3, 0.3)
*P* interaction	0.920	0.192	0.974	0.802	0.790	0.104	0.597	0.261	0.261
*P* for log-likelihood ratio	0.157	0.052	0.190	1.000	1.000	0.206	1.000	0.042	0.389

Age, sex, and race/ethnicity were adjusted both in model I and model II. P interaction is the value of p of the interaction test with sex as an effect modifier after adjusting for age, race/ethnicity confounding variables. APRI* and APRI** are two parts of the same data set. The result of APRI* and APRI** represents a curvilinear relationship between APRI and daidzein intake, with inflection points. MAFLD, metabolic associated fatty liver disease; CAP, controlled attenuation parameter; LSM, liver stiffness measurement; HSI, hepatic steatosis index; FLI, fatty liver index; FIB-4, fibrosis-4; NFS, NAFLD fibrosis score; APRI, AST to platelet ratio index; NA, not available.

Table 2 also showed the relationship between CAP, APRI, LSM, FIB-4, NFS, FLI, HSI, and daidzein intake by three different linear regression models. We found that CAP was negatively related to daidzein intake in the crude model. Similar results were present in the model I (after adjusting for age, sex, and race, *β* = −0.40, 95% CI: −0.66 to −0.14, *p* = 0.0024) and model II (after adjusting for age, sex, race, education level, marital status, PIR, smoking, and alcohol use, β = −0.37, 95% CI: −0.63 to −0.12, *p* = 0.0046). Stratified by quartiles of daidzein intake levels, trend analysis of the association between daidzein intake and CAP remained significant (*p* for trend = 0.0054). We also found a negative correlation between HSI, FLI, NFS, and daidzein intake, and the detailed results were shown in Table 2. In model I, the β values between daidzein intake and HSI, FLI, and NFS were −0.06 (95% CI: −0.10 to −0.03, *p* = 0.0012), −0.24 (95% CI: −0.37 to −0.11, *p* = 0.0003), −0.01 (95% CI: −0.01 to 0.00, *p* = 0.0005), respectively. In model II, the β values between daidzein intake and HSI, FLI, and NFS were −0.06 (95% CI: −0.10 to −0.02, *p* = 0.0027), −0.22 (95% CI: −0.35 to −0.09, *p* = 0.0008), −0.01 (95% CI, −0.01to 0.00, *p* = 0.0006), respectively. LSM was negatively associated with daidzein intake, but *p* > 0.05, with no significant statistical differences. The correlation between APRI, FIB-4, and daidzein intake was not strong (although *p* < 0.05, all *β* values were 0).

**Figure 4:**
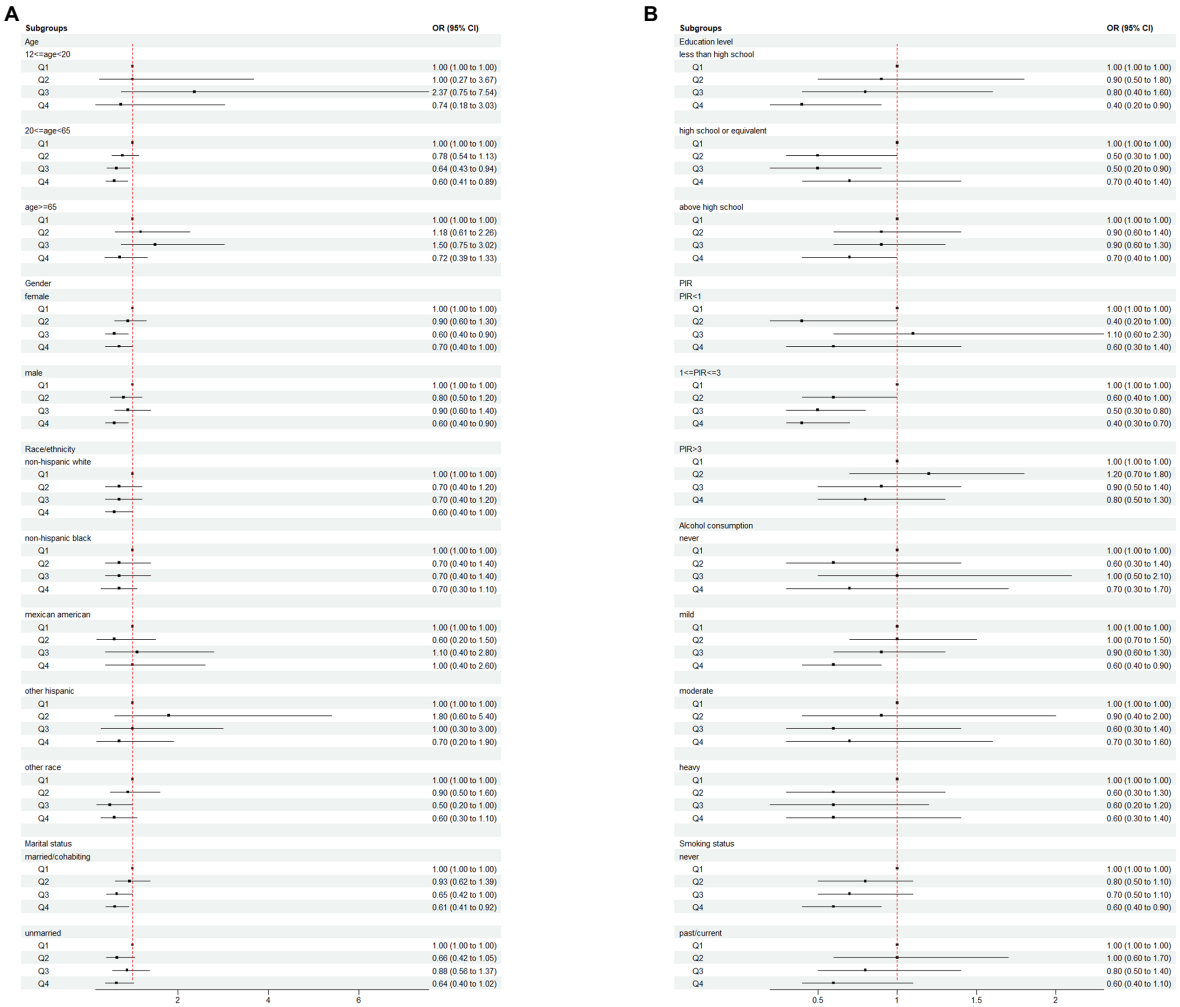
Subgroup analysis of the association between daidzein intake and the prevalence of MAFLD plotted in **(A)** and **(B)**. MAFLD, metabolic associated fatty liver disease; Q, quartile; PIR, family income-to-poverty ratio.

In Figures 2, 3 and Table 3, we further assessed the dose–response relationships between each outcome variable and daidzein intake by generalized additive modeling and smoothed curve fitting. With this dose–response relationship, we also used a log-likelihood ratio-based test to assess the presence of a saturation threshold effect and used a two-step recursive method to determine the inflection point of the smoothing curve. In Table 3, if the log-likelihood ratio test *p* > 0.05, it indicates a linear correlation between daidzein intake and the outcome variable, and the curve inflection point is not significant, referring to the results of Model I. If *p* < 0.05, it indicates a curvilinear relationship between daidzein intake and the outcome variable, and the inflection point is significantly present, referring to the results of Model II. Therefore, Table 3 and Figure 2 show a linear relationship between MAFLD, CAP, LSM, FIB-4, NFS, FLI, HSI, and daidzein intake, except for APRI, where the presence of two inflection points is a non-linear relationship. In addition, we performed interaction tests for gender and found that the *value of p*s for all interactions after adjusting for variables were >0.05, which indicated that the relationship between daidzein intake and each outcome variable was not significantly dependent on gender (Figure 3; Table 3). Figure 4 further stratifies by age, sex, race, education level, marital status, PIR, alcohol use, and smoking to determine the independent relationship between MAFLD prevalence and daidzein intake. We observed that higher daidzein intake was related to lower MAFLD prevalence among those aged 20–65 years, married/cohabiting, with less than high school level, with PIR between 1 and 3, and who never smoked (Figure 4).

## Discussion

A previous study from China supported a negative association between daidzein intake in isoflavones and the incidence of NAFLD (27). However, a number of growing evidence has supported that MAFLD diagnostic criteria are more useful than NAFLD for recognizing patients with a fatty liver at high risk of progressive disease (10–12). It was also necessary to study the association of daidzein intake with MAFLD in different ethnic groups. We found a relatively high proportion of daidzein intake among non-Hispanic white participants who had a high school degree or higher, were married/cohabiting, had higher household income, and did not have underlying conditions such as prediabetes, diabetes, and hypertensive disease. We also observed that the incidence of MAFLD decreased with increasing levels of daidzein intake and was not affected by gender, and more specifically, it is likely that this relationship was more significant among those aged 20–65 years, married/cohabiting, with education below high school level, with a PIR between 1 and 3 and never having smoked. We also found that CAP, HSI, FLI, and NFS were negatively associated with daidzein intake, but the correlation between daidzein intake and LSM, APRI, and FIB-4 was not strong.

The previous 2001–2004 NHANES study showed that income and race were associated with differences in dietary intake, particularly among individuals from low-income households and non-Hispanic blacks, who consumed less fruits, vegetables, grains, legumes, dairy, etc. (48). Another study from 2011 to 2016 NHANES data demonstrated the association of flavonoid intake in the US population with higher socioeconomic status (49). Our findings were similar, revealing a higher daidzein intake among non-Hispanic white, higher household income participants. Daidzein is protective against certain diseases related to estrogen regulation like prostate cancer, breast cancer, diabetes, and cardiovascular diseases (23–25). In addition, studies have shown that daidzein has the potential to prevent metabolic syndromes such as cardiovascular disease and diabetes (26), which may well explain the lower risk of diabetes and hypertension in participants with a high percentage of daidzein intake in this study. In mice, daidzein may ameliorate insulin resistance in obesity *via* direct modulation of hepatic *ab initio* adipogenesis and insulin signaling, and alter adipocyte metabolism to indirectly control obesity and reduce NAFLD by regulating adipokine expression through PPAR γ (50, 51). Another study also showed that daidzein improved lipopolysaccharide (LPS)-induced damage to hepatocytes by inhibiting inflammation and oxidative stress in mice (52). The MAFLD definition emphasizes more metabolic disorders and hepatic steatosis. Thus, the above-mentioned basic research further explains the negative correlation between daidzein intake and the prevalence of MAFLD.

A prospective cohort study from Guangzhou, China, included 2,694 participants and assessed the association between dietary flavonoid intake and NAFLD status using a food frequency questionnaire with face-to-face interviews, and revealed that higher flavonoid intake was related to a lower risk of progression of NAFLD in an elderly Chinese population (53). Our study also showed that the higher the daidzein intake in the middle-aged and elderly population, the more significant the reduction in the incidence of MAFLD. Another study from Tehran showed that education level and marital status were related to cardiovascular risk factors and dietary intake (54), and married women in the Hong Kong research had significantly higher intakes of vegetables, soy products, and fish than single women (55). Smoking was also considered an independent risk factor for poor prognosis in various chronic liver diseases (56–58). The result of a recent nationally representative cohort study from Thailand showed that smoking also increases the risk of all-cause mortality in patients with NAFLD, and this association was more pronounced in women with NAFLD (59). These studies also explained well that increased daidzein intake in the nonsmoking population in our study reduced the occurrence of MAFLD.

The pathophysiology of NAFLD/MAFLD has evolved from a “first strike” characterized by increased hepatic fat to a “second strike” consisting of adipokines, inflammatory cytokines, oxidative stress, and mitochondrial dysfunction; and the “multiple strikes” hypothesis consisting of insulin resistance, inflammation, lipotoxicity, cytokine imbalance, innate immune activation and microbiota disorders in the background of genetic and environmental factors (60–62). However, the accumulation of hepatic fat caused by insulin resistance remains the first and central striking factor (63). The current diagnosis of MAFLD is the basis of the presence of hepatic steatosis, which can be assessed clinically by non-invasive (blood biomarkers and imaging) and invasive means (liver biopsy) (64). Liu et al. (65). evaluated the accuracy of five commonly non-invasive hepatic steatosis algorithms such as FLI, HSI, Visceral Adiposity Index (VAI), NAFLD-liver fat score (NAFLD-LFS), and Steato text (ST) in the diagnosis of MAFLD using NHANES III, and the result showed that FLI had the highest diagnostic performance in the diagnosis of MAFLD. In this study, we also used three non-invasive liver fat assessment methods such as FLI, HSI, and CAP assess the relationship between daidzein intake and MAFLD, and the results showed that FLI, HSI, and CAP were negatively associated with daidzein intake, implying that daidzein may improve hepatic steatosis.

Given the current state of treatment for NAFLD/MAFLD, daidzein may be a potential therapeutic option. Recent research has demonstrated that soy protein concentrates and related isoflavones play an important role in lowering blood lipids, reducing hepatic steatosis, and improving the symptoms of NAFLD (66, 67). A cross-section study of 17,685 US adults also showed a negative association between the consumption of flavonoids and FLI (68). A randomized controlled trial from Iran found that 8 weeks of soy milk consumption and a low-calorie diet showed significant improvements in blood pressure and insulin resistance-related indicators in patients with NAFLD (69). Canadian food guidelines also recommend that the diet of soy foods or supplements may be a beneficial strategy to reduce the burden of disease and prevalence of MAFLD (70).

NAFLD can develop from simple steatosis to NASH, progressive hepatic fibrosis, cirrhosis, hepatocellular carcinoma, and liver failure (71, 72). NAFLD is related to increased mortality from hepatic and cardiovascular events, in which advanced fibrosis is considered an essential predictor of prognosis in NAFLD patients (72, 73). Liver biopsy is the golden standard for diagnosis of NAFLD fibrosis but is limited by the invasive nature of liver biopsy. Therefore, non-invasive assessments such as VCTE, point shear wave elastography (pSWE), magnetic resonance elastography (MRE), FIB-4, NFS, APRI, and other tests are used for screening fibrosis in NAFLD (74). In this study, we assessed the relationship between daidzein intake and hepatic fibrosis using four methods, including FIB-4, NFS, APRI, and LSM, and the findings revealed a negative correlation between NFS and daidzein intake levels, but no strong between daidzein intake and LSM, APRI, and FIB-4. There is insufficient evidence for the ability of daidzein to alleviate hepatic fibrosis. A previous study reported a significant inhibitory effect of high-dose soy isoflavones on thioacetamide-induced hepatic fibrosis in rats, which may be related to the inhibition of hepatic stellate cell activation and proliferation (75). The future may require us to investigate the association between daidzein intake and MAFLD-associated hepatic fibrosis in greater depth.

The advantages of our study are the use of representative national NHANES data, the multi-stage stratified sampling and the collection of relevant information by trained technical staff, the relatively large size of the sample included studies and the adjustment for multiple potential confounders to improve the reliability of the findings. However, our study also has some limitations. First, the cross-sectional study design of the study could not conclude whether there was a causal association between daidzein intake and MAFLD; therefore, further validation in a prospective cohort study is necessary. Second, the diagnosis of MAFLD in this study was defined as hepatic steatosis based on a CAP ≥248 dB/m measured by VCTE rather than by liver biopsy, which inevitably leads to a biased diagnosis of MAFLD. Also, non-invasive assessment methods do not perfectly predict the presence of hepatic steatosis and fibrosis in liver biopsies. Third, in our study, we tried to incorporate as many confounding factors as possible that were relevant to the study results, but we still could not completely rule out the possibilities of other confounding factors causing bias in the conclusions. Finally, due to limitations in the design of the USDA Food and Nutrient Dietary Study Database (FNDDS), it does not have data addressing the relationship between daidzein intake and calories, fat, protein, and dietary fiber. Therefore, we also do not know whether the daidzein intake is associated with a reduced intake of calories, fat, and protein. We will further investigate the relationship between daidzein intake and energy, lipid and protein metabolism, and dietary fiber, to elaborate more deeply on the relationship between daidzein intake and MAFLD.

## Conclusion

In conclusion, we found that the prevalence of MAFLD decreased with increasing daidzein intake and that CAP, HSI, and FLI were negatively correlated with daidzein intake, suggesting that daidzein intake may have improved hepatic steatosis. Therefore, dietary patterns of soy food or supplement consumption may be a beneficial strategy to reduce the disease burden and prevalence of MAFLD. However, the correlation between daidzein intake and hepatic fibrosis indicators such as LSM, APRI, and FIB-4 was not strong, and we may need to investigate the association between daidzein intake and MAFLD-related hepatic fibrosis more deeply in the future.

## Data availability statement

The raw data supporting the conclusions of this article will be made available by the authors, without undue reservation.

## Author contributions

ZY, YS, and QH conceptualized the study idea and conducted the interpretation, manuscript writing, and final approval. DG, XH, and FH performed the data analysis and collection, as well as the linguistic polishing. All authors contributed to the article and approved the submitted version.

## Funding

This work was supported by Jingzhou Science and Technology Bureau Plan Project (2021CC28-04 to ZY) and the Natural Science Foundation of Hubei Province (2019CFB567 to YS).

## Conflict of interest

The authors declare that the research was conducted in the absence of any commercial or financial relationships that could be construed as a potential conflict of interest.

## Publisher’s note

All claims expressed in this article are solely those of the authors and do not necessarily represent those of their affiliated organizations, or those of the publisher, the editors and the reviewers. Any product that may be evaluated in this article, or claim that may be made by its manufacturer, is not guaranteed or endorsed by the publisher.

## Footnotes

^1^ https://www.cdc.gov/nchs/nhanes/irba98.htm

^2^ http://www.r-project.org

^3^ www.empowerstats.com
