# Supplementary material for: Association between daidzein intake and metabolic associated fatty liver disease: A cross-sectional study from NHANES 2017–2018
Source: Front Nutr. 2023 Feb 13;10:1113789. doi: 10.3389/fnut.2023.1113789 (PMC9968739; doi:10.3389/fnut.2023.1113789)
Supplement: Supplementary file 1 [file Table_1.docx]

***Supplementary Table 1* Computational methods for non-invasive assessment models of liver disease**

| Scoring System | Algorithms |
| --- | --- |
| Fatty liver index (FLI) ^[37]^ | e^[0.953×ln（TG）+0.139×BMI+0.718×ln（GGT）+0.053×waist circumference -15.745]^/[1+e^0.953×ln（TG）+0.139×BMI+0.718×ln（GGT）+0.053×waist circumference-15.745^]×100 |
|  |  |
| Hepatic steatosis index (HSI) ^[38]^ | 8 × ALT/AST ratio+ BMI (+2, if DM; +2, if female) |
|  |  |
| AST to platelet ratio index (APRI) ^[39]^ | ([AST/upper limit of normal]/platelet count [10^9^/L])×100 |
|  |  |
| Fibrosis-4 (FIB-4) ^[40,41]^ | age ([year] × AST [U/L]) / ((PLT [109/L]) × (ALT [U/L])^1/2^) |
|  |  |
| NAFLD fibrosis score (NFS) ^[42]^ | -1.675+0.037×age (years)+0.094×BMI（kg/m2）+1.13×IFG/diabetes (yes=1, no=0) +0.99×AST/ALT ratio -0.013×platelet (×109/l) -0.66×albumin (g/dl) |

Abbreviations: TG, triglycerides; GGT, gamma glutamyl transferase; BMI, body mass index; ALT, alanine aminotransferase; AST, aspartate aminotransferase; DM, diabetes mellitus; PLT, platelet; IFG, impaired fasting glucose.

***Supplementary Table 2* Laboratory findings of participants by quartiles of daidzein intake (N=1476).**

| **Characteristic** | **Quartile 1** | **Quartile 2** | **Quartile 3** | **Quartile 4** | ***P* -value** |
| --- | --- | --- | --- | --- | --- |
|  | <0.02 | ≥0.02 to <0.18 | ≥0.18 to <1.55 | ≥1.55 |  |
| Fast glucose (mmol/L) | 6.34 ± 2.26 | 5.78 ± 1.19 | 5.89 ± 1.13 | 5.79 ± 1.18 | <0.0001 |
| Fast insulin (pmol/L) | 80.04 ± 96.10 | 72.52 ± 61.30 | 83.45 ± 60.04 | 78.42 ± 102.62 | 0.3389 |
| HbA1c (%) | 5.74 ± 1.00 | 5.57 ± 0.77 | 5.55 ± 0.76 | 5.55 ± 0.73 | 0.0064 |
| ALT (U/L) | 20.92 ± 12.84 | 22.12 ± 16.35 | 21.77 ± 15.13 | 21.07 ± 17.82 | 0.7079 |
| AST(U/L) | 20.38 ± 7.82 | 21.78 ± 10.00 | 20.94 ± 8.09 | 22.42 ± 14.52 | 0.0581 |
| TBIL (umol/L) | 8.28 ± 4.25 | 8.41 ± 4.07 | 8.64 ± 4.68 | 7.91 ± 4.94 | 0.1411 |
| ALP (U/L) | 76.97 ± 34.62 | 81.02 ± 44.02 | 84.65 ± 42.33 | 76.51 ± 34.70 | 0.0156 |
| Albumin (g/L) | 41.71 ± 3.26 | 41.50 ± 3.00 | 41.19 ± 3.16 | 41.48 ± 3.10 | 0.2082 |
| GGT (U/L) | 26.34 ± 33.88 | 26.60 ± 28.13 | 26.75 ± 34.31 | 22.93 ± 21.04 | 0.1818 |
| Creatinine (umol/L) | 77.63 ± 46.75 | 73.55 ± 19.72 | 78.38 ± 25.46 | 74.48 ± 15.95 | 0.0500 |
| Uric acid (umol/L) | 312.11 ± 79.09 | 326.38 ± 85.30 | 328.38 ± 87.62 | 306.39 ± 74.73 | 0.0002 |
| TG (mmol/L) | 1.43 ± 1.05 | 1.26 ± 0.72 | 1.24 ± 0.71 | 1.26 ± 0.63 | 0.0061 |
| TC (mmol/L) | 4.72 ± 0.98 | 4.92 ± 1.15 | 4.59 ± 0.96 | 4.88 ± 1.04 | <0.0001 |
| HDL-C (mmol/L) | 1.37 ± 0.36 | 1.46 ± 0.39 | 1.37 ± 0.38 | 1.45 ± 0.36 | 0.0007 |
| LDH (U/L) | 154.65 ± 26.42 | 152.06 ± 32.14 | 156.09 ± 28.33 | 157.26 ± 33.43 | 0.0931 |
| Hs-CRP (mg/L) | 3.14 ± 5.13 | 2.69 ± 4.55 | 2.85 ± 3.99 | 3.63 ± 9.01 | 0.1416 |
| WBC (10^9^/L) | 7.13 ± 1.99 | 6.96 ± 2.02 | 7.29 ± 2.01 | 7.46 ± 5.03 | 0.1440 |
| Hb (g/dL) | 14.18 ± 1.42 | 14.20 ± 1.31 | 14.29 ± 1.30 | 14.33 ± 1.20 | 0.3397 |
| PLT (10^9^/L) | 240.13 ± 60.46 | 241.53 ± 49.83 | 247.64 ± 59.58 | 239.54 ± 58.61 | 0.2049 |

Abbreviations: HbA1c, glycosylated hemoglobin; ALT, alanine aminotransferase; AST, aspartate aminotransferase; HDL-C, high-density lipoprotein cholesterol; TBIL, total bilirubin; ALP, alkaline phosphatase; GGT, gamma glutamyl transferase; TG, triglycerides; TC, total cholesterol; LDH, lactate dehydrogenase; Hs-CRP, high sensitivity-C-reactive protein; WBC, white blood cell; Hb hemoglobin; PLT, platelet.
